# Cyber insurance: state of the art, trends and future directions

**DOI:** 10.1007/s10207-023-00660-8

**Published:** 2023-01-16

**Authors:** Aggeliki Tsohou, Vasiliki Diamantopoulou, Stefanos Gritzalis, Costas Lambrinoudakis

**Affiliations:** 1 Ionian University, Corfu, Greece; 2 University of the Aegean, Samos, Greece; 3 University of Piraeus, Piraeus, Greece

**Keywords:** Cyber insurance, Cybersecurity threats, Underwriting process, Information security management systems

## Abstract

Society has become increasingly dependent on IT infrastructure and services. Additionally, the pandemic of COVID-19 forced the transition of the traditional way of working (i.e., physical presence) into a more modern and flexible one (i.e., working remotely). This has led to an increase of cyberattacks, as a direct consequence of the increase of the attack surface but subsequently also led to an increased necessity for the protection of information systems. Toward the protection of information systems, cyber insurance is considered as a strategy for risk management, where necessary. Cyber insurance is emerging as an important tool to protect organizations against cyberattack-related losses. In this work, we extensively examine the relevant literature on cybersecurity insurance, research and practice, in order to draft the current landscape and present the trends.

## Introduction

The increased dependency of modern society in digital services has led organizations in significant investments for administrative and technical countermeasures to prevent accidental or malicious cybersecurity incidents. Nonetheless, the realization of modern cyberattacks and cybersecurity incidents that result in severe impacts have made evident that organizational cybersecurity management cannot rely solely on risk mitigation measures [1]; instead, cyber insurance rises as a necessary complement to organizational safeguards. Exemplary cybersecurity attacks that resulted with critical severity include WannaCry and NotPetya in 2017, which affected thousands of companies in multiple regions and industries [2]. Another example is the ransomware attack that affected governmental organizations in the USA (i.e., Departments of Defense, Homeland Security, State, Treasury, Energy and Commerce, as well as several others) [3]. Advanced cyber threats of high severity that prevail today include cryptojacking, malware, supply-chain attacks, ransomware, business email compromise and others [4, 5].

Information security management is widely accepted as a risk-based process [6]. Following risk assessment, organizations can decide how to manage risks by choosing amongst four strategies: risk modification, risk retention, risk avoidance and risk sharing [7]. The latter strategy (a.k.a., risk sharing) pertains that the organization shares the risk with an external party that can most effectively manage the particular risk depending on risk evaluation. Risk sharing can be implemented using insurance to support the consequences of an incident, or by sub-contracting to prevent the risk from materializing. Cyber insurance market and practices are rapidly growing and expected to further develop [2, 5]. Despite the strong motivation that organizations have to employ insurance as a cybersecurity strategy for specific threats, as the same time they are reluctant to do so [8]. Given that the cyber insurance domain is going through foundational development, transformation and shaping, in this paper we aimed to investigate and present an overall view of the domain today, including drivers, obstacles, practices and involved processes, status and involved stakeholders. Similar literature reviews have been performed by few researchers, including [9, 10] and [11]. Our work not only updates the insights that these works provide, but it is also complementary to their findings. Specifically, the authors in [9] discuss the stakeholders of cyber insurance market, aspects regarding the cyber insurance process, policies and contracts. The author in [10] particularly focuses on the challenges faced by the insured (e.g., the complexity of the insurance contract). In [11], the authors also highlight practical challenges, including how to assess cyber risks during the underwriting process and how to calculate and receive appropriate compensation. Our purpose in this article is to bring together the insights given by past literature analysis works and perform an extensive and updated literature review on the current cybersecurity insurance research and practice toward drafting the current landscape and providing insights on future directions. In addition to the approach taken by past literature analyses, in this paper we place special attention to recent industrial survey reports and statistical data, to reveal the most prevailing and up to date information regarding the cyber insurance market. Our findings deriving from the analysis of existing literature highlight the distinguishing characteristics of the cybersecurity insurance market, including the dominance of large clients, the complex and lengthy underwriting process compared to other insurance products and the imbalance between demand and capacity. Our analysis also presents the available types of insurance policies and the typically insurable cybersecurity risks. Nonetheless, insurance policy risk coverage limits are not as clear as in other insurance products; for example, an incident might be detected after some time, which makes challenging for the insured to receive coverage [9]. Finally, our work aggregates information related to the underwriting and claims management process, which can be informative for organizations who wish to consider the option of insurance for cybersecurity. Further, this literature review recognizes research and practical directions for further development of the field toward addressing identified challenges and obstacles.

The rest of the paper is organized as follows: In Sect. 2 the literature analysis methodology that we followed is presented. Section 3 presents the most prevalent cybersecurity threats and trends and indicates how these threats take the role of the insurance drivers. Section 4 analyzes the various characteristics of the cyber insurance policies and their association with the different cyber incidents. Next, Sect. 5 describes the underwriting process and presents the claim insurance preparation, highlighting the important role of the Information Security Management System—ISMS of a business. Section 6 gives insights from the cybersecurity insurance market, while Sect. 7 concentrates the cyber insurance trends, research and practical directions. Finally, Sect. 8 concludes the paper by raising issues for future research.

## Literature analysis methodology

For the literature selection and analysis, we followed the guidelines proposed by [12] and [13]. Specifically, we followed the steps “Frame the question and choose appropriate methods steps,” “Identify relevant work,” “Extract relevant data on outcomes and quality,” “Summarize the evidence” and “Interpret the evidence.”

For the first step our research question is “what are the drivers, obstacles, practices and processes, status and involved stakeholders in the domain of cybersecurity insurance?”. Regarding the sampling protocol, we have specified inclusion and exclusion criteria, in order to identify the studies that provide evidence about our research question. Specifically, we included both academic studies and industrial reports, to capture the status of the field as represented by scientists and practitioners. The keywords for our search included the terms “cybersecurity insurance” and “cyber insurance,” and we narrowed our search on studies that have been published in the last decade, given that we aim to provide insights on the current trends and challenges. Regarding exclusion criteria, we excluded studies that have not been accepted for publication (e.g., draft works) or are the result of a thesis.

For the second step, we selected the databases and conducted a search using the chosen keywords. Our selected data sources included the leading computer science and information security Journals and Conferences: Computers & Security, Journal of Information Privacy and Security, IEEE Security & Privacy, International Journal of Information Security, Telematics Informatics, Information Technology and People, Information and Computer Security, AIS. Additionally, we searched the databases of Scopus, Web of Science, ERIC, Science Direct, Google Scholar, IEEE and the digital libraries Springer Link, ACM Digital Library, Elsevier. Further, for industrial reports we included the digital libraries of the professional communities of LinkedIn and Research Gate. We also included reports and standards published by international expert organizations, such as the European Union Agency for Cybersecurity (ENISA) and the International Standardization Organization (ISO). Finally, we included yearly survey reports by globally acknowledged information technology and insurance organizations, such as Deloitte, Allianz, Federation of European Risk Management Associations. After applying the keywords in the data sources, the initial sample of studies was selected. Then, we conducted backward reference searching by applying the inclusion criteria on the references of the selected studies.

For the third step, we provided the analysis of the literature section. Following the analysis of the literature, certain themes were inductively extracted for our understand and for the insights that we gained on the domain: cyber insurance drivers, cyber insurance policies, cyber insurance involved processes (and especially the underwriting process) and topics pertaining the cyber insurance market (e.g., stakeholders, obstacles). We then analyzed the selected studies with relevance to these themes. Regarding the fourth step, we provide a discussion section in which we combine the results of the studies into further presenting cyber insurance trends, research and practical directions.

For the final step, we elaborate on the implications of our findings and provide our views on the strengths and weaknesses of our literature review in the conclusion section.

## Current cybersecurity threats and trends as insurance drivers

In this section, we present the prevailing cyber threats which according to reports [4, 5] are challenging for organizations and push them to seek for cyber insurance coverage, including supply-chain attacks, ransomware, business email compromise and funds transfer fraud.

### Ransomware

Ransomware is a type of malicious attack in which attackers encrypt an organization’s data and demand payment (ransom) to restore access. Ransomware has been assessed as the prime threat for 2020-2021, with several high profile and highly publicized incidents [4]. In advanced forms, ransomware evolved in double extortion in which the criminals blackmail the victims not only for restoring the encrypted database, but also for preventing them to leak the data in the public [14]. Researchers and reports highlight a continuously increased rate of ransomware attacks in 2020 and 2021 [4, 15]. Together with the increased rate of ransomware attacks, it is also noted that the ransom amount requested keeps increasing [2, 16]. Further, in 2020 and 2021 the Ransomware-as-a-Service (RaaS) business model is blooming. Ransomware-as-a-Service is a business model that allows inexperienced attackers who do not hold sophisticated technical skills to efficiently orchestrate attacks. This is even further facilitated by the availability of ransomware packages in the Dark Web [4, 14]. To multiply the impact of their attacks, cybercriminals target managed security service providers, who function as providers of software for several organizations; thus, with a single successful attack they affect hundreds - even thousands - organizations. The average recovery time from ransomware is approximately 19 days, which leads to significant business interruption. The severe impacts of ransomware include loss of reputation, revenue, money and data [16].

### Business email compromise

Business email compromise (BEC) is an attack in which the malicious actor succeeds in getting access to an employee’s email account and subsequently utilizes this access for further unauthorized actions (a.k.a., appearing as authorized). Thus, BEC can eventually lead to a wide range of losses, including ransomware, funds transfer fraud, data breaches and others. As an indication of the consequences of BEC attacks, it is recorded that the cost of BEC attacks in the United States was approximately 1.7 billion dollars in 2019 [17], and the average cost per BEC attack was 30 thousand dollars in 2020 and 50 thousand dollars in 2021 [18]. A significant rise of BEC attacks is recorded across regions and industries particularly in 2021 compared to 2020 [3, 19, 20]. The conditions that were enacted by the COVID-19 pandemic contributed to the increase of BEC incidents, given the lack of physical presence and interpersonal communication, which frequently were replaced by email communication.

### Funds transfer fraud

Funds transfer fraud (FTF) breaches refer to attacks that aim to manipulate authorized users or activities toward wiring funds to unauthorized recipients. FTF is commonly realized subsequently to other attacks, such as ransomware and social engineering. While other advanced attacks, such as ransomware, require more sophisticated techniques, tools and knowledge, FTF is often perpetrated through BEC and social engineering, and thus it is easier to be realized. Nonetheless, FTF can result in significant losses; indicatively, the average amount of losses per FTF attack in 2021 is 326 thousand dollars, which is significantly higher than 2020 in which it was 124 thousand dollars [19].

### Supply chain attacks

The past years, organizations have made digital transformations which were commonly realized through outsourcing services, such as hosting by cloud computing providers. Those providers are in turn reliant on other service suppliers and so on. Obviously, by relying on external vendors (and their vendors and their vendors) organizations have become exposed to higher risks outside their control. Consequently, individual cyber risks have been transformed into supply chain risks, since one successful attack can simultaneously affect multiple organizations due to the creation of complex supply chains that support the market today [19]. Malicious attackers increasingly target software vendors and service providers, knowing that significant number of organizations rely their infrastructure on them, to achieve multiple casualties with a single attack.

### Effects of the COVID-19 pandemic

The conditions that emerged due to the COVID-19 pandemic have enabled significant rise in cyberattacks. Organizations were forced to rapidly alter work conditions and allow massive teleworking in order to ensure business continuity, and at the same time receded in cybersecurity protection since they didn’t have the time to make necessary cybersecurity preparation [21]. Employees started working from home by involving own equipment and networks that have not been customized according to organizational security policies, and thus were vulnerable to cybersecurity threats [3]. Further, several organizations relied on protocols, like the Microsoft Remote Desktop Protocol (RDP), which became convenient attack points for cybercriminals [22]. On top of the technical vulnerabilities that were enabled by the pandemic, the isolation of employees prevented interpersonal communication with one another and with information technology support employees. This hindered the promotion of cybersecurity culture and awareness, making employees more vulnerable to social engineering and other related attacks [2], as well as the timely incident report and handling.

## Cybersecurity insurance policies

ISO 27102 [23] defines a cyber incident as a cyber event that involves a loss of information security or impacts business operations. In [10], the author classifies cyber incidents into five types: system malfunction, data breach, data integrity or availability loss, human errors and malicious activities. Cyber insurance can compensate the insured against potentially significant financial losses associated with a cyber incident; the insurer underwrites risks by accepting liability and guaranteeing payment to the insured in case loss or damage occurs.

### Incidents covered by insurance and eligibility criteria

Cyber insurance may cover a variety of cyber incidents that can lead to financial losses, business interruption, network damage, etc. Among the cyber incidents that can be covered by insurance are system malfunctions, data breaches, loss of integrity or availability, malicious activities and human errors [23]. Ransomware is one of the top cyber threats that drive organizations to select insurance as a treatment strategy [24]. According to Cloudian survey, organizations received compensation for the ransom paid (approximately 59% of the ransom amount) and for other losses resulting from the ransomware attack (approximately 58% of the losses).

Nonetheless, research notes a number of discrepancy areas which contribute to lack of clarity and to the limited adoption of insurance as a treatment option [1]. The first discrepancy is whether non-malicious events (e.g., mistakes, omissions) are covered, and which events are not covered (e.g., power outages). The second discrepancy concerns the extent to which events at sub-contractors or extern service providers are covered and insurance companies take different approaches on this matter. Some insurance companies cover events that occur at external collaborations, but they offer this option only for a list of named providers (i.e., commonly reputable vendors). The third discrepancy refers to the degree in which insurance covers subsidiaries and corporate entities in different jurisdictions or new subsidiaries created during the policy period. Given these discrepancies, it is significant to establish insurability criteria that can specify the conditions under which a risk is insurable [9]. This is important because predictions about a risk can become more accurate when the risk satisfies certain criteria. There have been studies ([25–27]) that have highlighted the need to establish such criteria so that the insurance process becomes more reliable and, consequently, cyber risk can be more easily insured. To this aim, various studies (e.g., [28]) have focused on establishing these criteria and investigating the insurability of cyber risk [26]. As discussed in these works, a number of problems with the insurability of cyber risk impede the market development. Therefore, researchers and practitioners realized the importance of establishing these criteria to overcome these problems with the aim of helping market grow, increasing insurance risk pools and capacity, boosting market competition and, ultimately, keeping prices down. The authors in [9] summarized these criteria, which we present in Table 1.

**Table 1:** Insurability of cyber risks (based on [9])

**Insurability criterion**	**Description**
Mehr and Cammack [29]	
Incidental loss	The incident must be fortuitous and not under control of insured
Limited risk of catastrophically large losses	Catastrophically large losses must happen with very low frequency
Calculable loss	It must be possible to estimate or calculate possible losses and probability of an incident
Large number of similar exposure units	A large number of homogeneous exposure units must be available to facilitate the probability determination
Affordable premium	The premium must be reasonable for the insured
Definite loss	The loss must be difficult to forge. Its time, place and cause must be easy to determine
Large loss	The losses must be large enough for the insured to be born by himself/herself
Berliner [28]	
Randomness of loss occurrence...	...incidents must happen independently.
Maximum possible loss...	...per incident should be manageable for insurer.
Average loss per incident...	...should be moderate.
Loss exposure...	...should be large enough.
Information asymmetry...	...should be too high.
Insurance premium...	...should be affordable for the insureds.
Cover limits...	...should be suitable for insureds.
Public limits...	...should be respected.
Legal restrictions...	...should not be violated.

### Cyber insurance policies

The cyber insurance terms are documented in a cyber insurance policy, which can be a stand-alone policy or can be included as a part of other organizational policies (e.g., general liability policy, property policy) [23, 30]. Therefore, a cyber insurance policy is a contract between an insured and an insurer which defines terms, conditions and exclusions for the insured risk. The insurance premium is a fee paid by the insured to the insurer for assuming the risk. Policy exclusions are losses that are excluded from the policy and may refer to bodily injuries and property damage, terrorism threats, intellectual property violations, loss of reputation, or others. The authors in [31] refer to the most common exclusions in cyber insurance policies, including criminal or fraudulent acts, infringement of patents, negligence disregard for computer security, IP theft, acts of terrorism, war or military action, bodily injuries. The insurance may comprise coverage limits, which may refer to a waiting period or a deductible amount, that the insured should pay before a claim can be made.

The insurance coverage may include a broad range of cybersecurity threats that can cause business interruption, liability costs, legal penalties incidence response costs or other forms of impact and varies significantly between different insurance products. Coverage may differ depending on regulatory limitations, market practices, insurer business strategy and the needs of the insured. However, there is research [31] that has analyzed coverages and applications (in the USA) that see no actual differences in policies between markets and overall identified strong similarities across covered losses, which suggests that carriers have a certain amount of confidence in their ability to price these risks.

### Types of policies and coverage

Two types of insured coverage exist: first party and third party. The first-party coverage insures against the losses for the insured itself, while the third-party coverage covers damages to third parties [9, 11, 31]. By combining findings from previous works, we present in Table 2 losses that are covered in the two types of coverage.

**Table 2:** Types of coverage by insurance policies (based on [9, 31–33])

Type of coverage	Coverage	Indicative cases
First party	Loss or damage to digital assets	Coverage of costs to repair or restore lost or damaged data and software (e.g., computer attack or data compromise response)
	Business Interruption	Coverage of lost income and costs due to business interruption as a result of computer network failure
	Cyber extortion	Coverage of forensic investigation costs, coverage of ransom payments
	Forensics investigation and Restoration costs	Coverage of costs to investigate and contain the data breach and to restore systems and networks
Third party	Credit monitoring/ Call Center	Coverage of expenses for credit monitoring program offered to customers affected by a data breach and costs of call center services to answer to customer inquiries
	Multi-media liability	Coverage of costs that relate to the infringement of intellectual property rights and distribution of materials
	Public relations	Coverage of costs for protecting and restoring reputation and public image
	Security and privacy breaches/Fines and Penalties/ customer notification costs	Fines and penalties resulting from noncompliance with personal data protection regulation or breach of third-party business information

## Cyber insurance preparation and underwriting process

For the insurance company to decide about assuming the insured risk and formulate the respective policy, there is a process which is commonly referred to as the underwriting process. The underwriting process typically involves some preparatory activities to assist in determining whether to accept the risk and to determine an adequate premium for the risk coverage, including acquiring information about the insured’s cybersecurity practices, assessing the insured’s cyber risks, assessing an insurer’s business risks, determining the insured insurability and price; and developing the cyber insurance policy [23].

### Information collection

For the insurance company to perform the underwriting process, the potentially insured organization needs to provide to the insurance company access to information that will assist the formulation of the insured risk profile [23, 31, 34–37], including:

- Business profile and mission statement
- Key stakeholders (information about clients and providers)
- Types of data under processing
- Details of information systems and any outsourcing agreements
- Details of an ISMS
- List of existing countermeasures
- Record of past cyber incidents
- Reports related to security management, such as audit reports
- IT security budget and spending
- Information about past and existing insurance coverage
- Financial records and others

However, according to [31], the different insurance provider may pay different attention to the above requested information. Most providers place emphasis on the amount and type of data that are processed by the applicant organization, while less emphasis may be placed on other information (e.g., the technical and business infrastructure, the stakeholders, the IT security budget). Insurance experts state that they also pay attention to informal and less quantified information, such as the insured responses to questions about organizational security management practices [35]. The experts say that they consider if the representatives of the insured respond based on accurate and updated data. Further, they consider if the associated collaborators that the organization involves in security processes (e.g., incident management team) are experts.

The insurer assesses cyber risks of the interested organization to assist in determining whether to accept covering the risk and to determine an adequate premium. The risk assessment examines both the risk exposure of the interested organization and the status of in place security controls. As part of the underwriting process the insurer also considers risk accumulation scenarios, i.e., events that can result in claims across a large part of the portfolio of insurance policies (e.g., electricity outage in a large geographical area).

### The role of ISMS as source of information for the cyber insurer

As mentioned in Introduction of this section, the process for developing a cyber insurance policy includes a series of preparatory activities, to determine whether the cyber insurer will accept the insured’s cyber risk or not (ISO 27102 [23]). After collecting information as described in Sect. 5.1, a cyber insurer is able to gain useful insight of an organization from information that derives from the documentation of an organization’s ISMS. According to ISO 27102 [23] an ISMS that aligns with ISO 27001 [6] can be the core source of information for the underwriting process, as well as the life cycle of the insurance policy. Table 3 enlists documentation which is produced by the ISMS and is meaningful for the insurance underwriting process.

**Table 3:** ISMS as a source for the underwriting process (based on ISO 27102 [23])

**ISMS stage**	**Documentation**
Planning	ISMS scope
	Security objectives
	ISMS policy
	Risk assessment method
	Statement of Applicability
Operate	Documentation of information processing activities
	Risk assessment results
	Risk treatment plan
Performance Evaluation	Monitoring and measurement results
	Audit results
	Management reviews results
	Nonconformities and any subsequent actions taken
	Corrective actions
Support	Stakeholders’ competence
	Awareness programs

The ISMS can be a valuable source for the insurance company to gain insights for the cybersecurity risks profile, the security management processes of the organization and the existing countermeasures, past incidents, corrective actions, etc. The ISMS is valuable also for establishing a standardized communication format between the insurer and the interested insured.

### The claims management process

Although the cyber insurance claim process may vary from one company to another, the typical process includes^1^:

1. The insured identifies a cyber incident that falls under the policy coverage
2. The insured contacts the insurance company
3. The insured contacts the legal advisor, who will collaborate with the insurance company and direct the technical team (e.g., forensics investigators) or the insured contacts an incidence response firm (combining legal, forensics and other services) [38]
4. The forensics team performs an investigation to reveal the events associated with the incident
5. The crisis communication team coordinates internal and external communications to minimize reputation damage and comply with notification requirements
6. The incident recovery team works toward bringing the systems back to normal operation
7. The insured collaborates with the insurance company to leverage coverage

According to research, however, the claims management process may involve challenges [9, 11]. The first challenge refers to the time of the claim, since several attacks may remain undetected, and the breach may be noticed long time after the incident. In this case it becomes challenging for the insured to receive coverage. A second challenge refers to the necessity to perform and document a forensics investigation as a pre-requisite to submit and validate the claim. This imposes additional burden on the insured and involves communication with several stakeholders, thus the organization may not be able to maintain the secrecy of the incident which can damage the business reputation. The insured may consider this before making a claim, especially if the coverage is borderline and therefore it is uncertain if it will be achieved. However, the necessity to reveal confidential information as a challenge varies depending on the way that the stakeholders are involved; if the legal advisors coordinate the forensics team, the client-attorney privilege may cover the secrecy of the investigations and thus the forensics results may not be formally documented (i.e., the insured will not submit a forensic report with the claim) or may be reported only orally [38, 39]. Nonetheless, such lawyer practices have been strongly criticized as they substantially impair the insured ability to learn from cybersecurity incidents and implement long-term remediation efforts to prevent cyberattacks and incidents.

## Cybersecurity insurance market

Cyber insurance market is growing in rapid pace and the predictions suggest further development within the next years [2]. According to surveys [40, 41] the global cyber insurance market was estimated at 4.85 billion dollars in 2018, at 6 billion dollars in 2019 and is predicted to reach 15 billion dollars until the end of 2022 and 28.6 billion dollars until 2026.

### Distinguishing characteristics of the cyber insurance market

Research recognizes some distinguishing characteristics of the cybersecurity insurance market. First, the market is dominated by customers that are large companies [1]. One reason for this is that large companies are often partners/customers of other large companies which consider cyber insurances a prerequisite for collaboration (i.e., a sine qua non for doing business). Further, large companies typically commission insurance intermediates who disseminate emerging cyber threats and diffuse cyber insurance products. Smaller companies are less likely to be exposed to respective information and thus they are less familiar with cyber insurance as a risk treatment strategy.

A second distinguishing attribute refers to the complexity of the underwriting process. The underwriting process in cyber insurance is more complex and longer compared to other (traditional) insurance products [1, 10, 11, 34]. This is mainly because the cyber insurance coverage and conditions are not as standardized as other insurance products. Another factor contributing to the complexity of the underwriting process is that cyber insurance is not a single/standard product; instead, it is highly tailored to each insured organization. Further, in most cases cyber insured organizations are first time clients, and thus the process to identify and quantify cyber risks which the insured wishes to transfer is rather difficult since it is a first-time exercise. For other insurance products, the market is more mature, especially because clients had the time to change from one insurer to another and thus have performed the underwriting process several times. However, it is important to mention that although the bulk of the market moves in a slow bureaucratic rate, there are also new business models emerging, like Coalition^2^ or AtBay^3^, who try to transform the way we think about risk. They take a fundamentally new approach to ensuring cyber risk by assessing an organization’s risk when they apply for insurance and proactively monitoring and alerting the organization to prevent risk before it is realized. Thus, the process becomes simpler.

Finally, research recognizes a significant imbalance between the demand and capacity in the cyber insurance market. Although the demand of cyber insurance products continuously increases, the prices of insurance premiums keep increasing as well, because more and more organizations find difficulties to internally handle cybersecurity risks and seek for insurance solutions. According to Howden Cyber Insurance Survey [2] and the Council of Insurance Agents Brokers [42] the demand of cyber insurance products has increased significantly the last six months of 2021; nonetheless there is difficulty of the market to satisfy this demand. Based on the reports, the main reason for the imbalance between the demand and the market capacity is the complex and long underwriting process, which basically includes customized insurance products per insured organization.

### The role of the cyber insurance providers

Although cyber insurance is a method for the insured to transfer risk to an external party, cyber insurance companies are doing more than receiving the risk on behalf of the insured. The activity of the insurance providers is not limited to that, they are actively managing the underlying cybersecurity risks [33, 61]. Cyber insurance companies are actively engaged in assessing installed countermeasures, proposing controls and processes for loss prevention and offering first response to cyber incidents. In this way they fill internal organizational roles, such as legal, compliance, information technology and crisis management positions. According to researchers [1], cyber insurers confirm that first response services are vital part of insurance products. Incident response services are commonly offered as an one-stop-shop call service to the insured, and they offer it in collaboration with other partners, such as information technology experts, lawyers, public relation consultants, etc. However, some in the literature [38] have criticized the extent to which cyber insurance companies strongly influence global diffusion of cybersecurity protection and increase cybersecurity mechanisms. Cyber insurance has been disappointing regarding the diffusion of protection, but seems to contribute by offering post-breach services. Cyber insurance market rarely includes basic security procedures in contracts and gives no actual motives for organizations to invest in cybersecurity. They seem to believe that covering expenses post incident is more effective than mitigating risks in advance.

### Cyber insurance claims

Surveys and reports demonstrate that the cyber insurance claims from insured organizations are rapidly growing; according to the data presented by the insurance company Allianz [43] in 2016 the company handled approximately 100 respective claims while in 2020 the respective claims were about 1050. Similarly, Howden Cyber Insurance Survey [2] reported that claims associated with both first-party and third-party policies increased radically the past 5 years. NetDiligence [44] also stated that 5.797 claims were received between 2016-2020 out of which 30% occurred in 2019 and 25% in 2020. According to the reports [19, 44] most claims aim to address ransomware attacks, hacking, BEC, FTF, phishing, malware, and others. The most expensive claims relate to ransomware (312K dollars for 2020 and 184K dollars for 2021) and FTF (152K dollars for 2020 and 247K dollars per claim for 2021) [19]. In terms of industry sectors, most claims were submitted by consultancy/professional services, health care, financial services, manufacturing, retail and others.

### Obstacles for the market growth

Despite the rapid growth of the market, organizations are reluctant to adopt insurance as a risk treatment strategy; organizations aim in priority first for risk avoidance, then risk minimization, risk transfer and risk acceptance [45]. This reluctance may be to some extend understandable, when taking into consideration the fact that several organizations (approximately 35% [46] state that they are not satisfied with their insurance and cyber claims management.

An obstacle preventing the growth of cyber insurance and distinguishing cyber insurance market from other insurance markets is the correlation that appears between cybersecurity risks [47]. For providing stable claims management conditions an insurance company must maintain a sufficiently large pool of insured organizations and cover risks that are relatively independent and uncorrelated. However, in the case of cyber insurance, risks appear to be correlated and interdependent [48–51], which is not the case for other insurance markets and products [45]. Cybersecurity risks are correlated because the same event can affect multiple organizations/systems simultaneously: the fact that the different computer systems are designed and implemented in similar way and through reliance on the same vendor (e.g., Microsoft Windows) makes most systems vulnerable to the same event. Additionally, cyber security risks are also interdependent because a compromised system can impair risk to other systems (e.g., malware) because they might have been developed relying on a same development library or protocol (for which a vulnerability has been exploited) [52, 53]. Furthermore, businesses around the world rely on the same small number of systems (e.g., Symantec) for downloading virus definitions lists and IPS/IDS signatures; if one of these lists/systems gets compromised, all businesses (and thus insurance companies) may find themselves in trouble. Also, in contrast to the physical world, where risks are geographically dispersed, in the digital environment the exploitation of the network results in the rapid spread of any attacks (such as viruses and worms) across all geographical boundaries. As a result, a single event is likely to incur concurrent claims from many insured organizations. Finally, cost is another important aspect/obstacle; insurance contracts tend to be overvalued because insurers are unable to anticipate secondary level customer losses, such as reputation damage [54].

## Cyber insurance trends, research and practical directions

Cyber insurance is a growing field with several open research and practical challenges. One of those challenges is the harmonization of language and terminology used among the insurance stakeholders, which is expected to act as a driver for the maturity of the field and the expansion of the market.

### Harmonization in language and underwriting process

The underwriting process involves several mechanisms that the insurers use to collect information from potential customers, including questionnaires, meetings, desk research, audit reports and risk assessment reports. Nonetheless, the most prevalent mechanism is the questionnaire [1, 31, 32], which is used to collect quantitative and qualitative information on the underwritten risk. According to a study of ENISA [32] from ten leading insurance carriers, there is a lack of harmonization in the risk assessment language that is employed across insurers to draft the underwriting questionnaires and the risk coverage incorporated in the insurance policies. The lack of harmonization may lead to disconnect the link between cybersecurity standards, cyber insurance and underwriting information, which affects the ability to determine loss correlation. A lack of connection between the critical threats and exposure and the provided coverage may lead to large uninsured incidents or high percentage of non-covered claims.

Currently, ENISA [32] reports that multiple cybersecurity standards are used to demonstrate compliance to best practices (e.g., COBIT, ISO 27001, ISO 27002, NIST), and therefore, an organization may face different risk assessment methods and questionnaires by different insurance carriers during the underwriting process to define risk exposure. Therefore, *a first potential research perspective is the harmonization of the risk assessment language, regardless of the standard that is used*, so that insurers can have similar points of reference to assess the risk profiles of potential insured organizations. However, even when the same risk assessment standard is used, two insurance providers may ask different questions to assess the existence and exposure to the same risk. Cyber insurance market is less mature and standardized compared to more mature insurance products (e.g., car insurance) for which different insurers will ask the same questions to assess a buyer’s risks. According to the study that ENISA [32] conducted, the questions utilized by ten large insurers were significantly variant (i.e., different questions per carrier, different definitions for similar risk areas, overlapping questions for key risk areas). Further, the questionnaires were found to be without alignment with the security standards. Therefore, *a second potential research direction refers to the harmonization between security standards and underwriting questionnaires*. Finally, the same report states that there is heterogeneousness in the language used to describe insurance coverage (i.e., the wording that the insurers used to describe the different coverage types they offer). Using simple and clear language to describe coverage and policies is encouraged to prevent ambiguities and misunderstandings, to facilitate the comparison of policies and to enable to consistency in the treatment of claims across the insurance industry [55]. Thus, *a third avenue for further development is the harmonization of policy and coverage language*. The harmonization in this aspect will allow customers to be able to compare prices and coverage that the various insurers offer.

### Familiarity with the underwriting and claims management processes

According to research studies, a significant obstacle preventing the growth of cyber insurance is the complexity and lengthy duration of the underwriting process [1, 34], which is attributed to the lack of standardized coverage, policies, conditions, etc. Most organizations encounter the cyber insurance process for the first time and most insurers tailor the underwriting process per customer [1]. The complexity of the process is amplified by the lack of statistical and actuary information [11, 56], the constant evolvement of the threat and risks landscape, and the specialized knowledge required by insurers and insured organizations to understand the threats, the exposure, the impacts and the related countermeasures. Further, there exist challenges pertaining risk prediction and simulation, such as how to measure security posture, how to calculate the attack surface, how to consider the organizational characteristics (e.g., size, sector, reputation), and how to take into account risk that results from third-party relations [11]. Although technological tools that support and assist the underwriting process are gradually becoming available, *there is a need to advance the technological tools which can facilitate the insurers and insured organizations* [57]. For example, for the insured organizations it is beneficial to advance risk quantification tools for the evaluation of estimated aggregated loss and estimated probable loss and benchmarking tools to assist insurers the comparison between the company’s security posture level to other similar companies. For the insured organizations it would be beneficial to advance the preparatory technological tools, such as simulation tools of the insurance processes (e.g., underwriting process, claim submission process, structured repositories of the information to be shared, templates and formats of the commonly requested information). Although the underwriting and claims submission processes vary per insurer and potential insured company [1], the provision of simulation environments will enable the familiarity of organizations, allowing faster response to inquiries and information exchange during the actual processes.

### Cyber incidents databases and big data analytics

The contribution of big data analytics tools and techniques (e.g., data mining, cluster analysis, machine learning) for improving cybersecurity posture of organizations has been widely studied [58]. Some of the areas in which big data analytics techniques have contributed to cybersecurity enhancements are intrusion and anomaly detection, spamming and spoofing detection, malware and ransomware detection [59], code security, cloud security, etc. Big data analytics and machine learning could be essential tools also for cyber insurance [60], such as for developing cyber risk prediction models to design insurance products or to allow insurers to early notify policy holders for predicted attacks and recommend immediate actions in the appearance of risk patterns among policy holders. Overall, according to [56], big data techniques, artificial intelligence and emerging technologies have the potential to transform the way insurers perform the underwriting process, calculate premiums and estimate risk posture. Nonetheless, a barrier for exploiting the advantages of big data analytics is the lack of historical and actuarial data to enhance reliability and remove uncertainty from cyber risk assessment and price calculation. Currently, insurance underwriters and brokers, rely on expensive, commercial, third-party databases developed by data providers that compile information on cyber incidents and losses. According to [56], cyber insurers today rely on three to four major data brokers. These databases contain records from publicly available sources about cyber events and different types of cyber risks. The events can be classified by company type and size, industry type and revenue amount. Further, the data include information about the events, such as the number of records affected, the type of losses suffered, how the breach occurred and the type of cyber risks posed. Nonetheless, the capacity of these technologies is strongly reliant on the reliability, quality and completeness of the sample data and thus today it is hindered by the lack of sources of complete information, while the disparate sources contain different types and amounts of data. Therefore, the cyber insurance market would strongly benefit by advancements that can battle the limitations and deficiencies in the source data, such as through *the creation of anonymized cyber events and incidents repositories*. For example, ENISA [32] recommends the creation of a European Union wide repository of incidents to provide aggregate data from multiple sources. For this objective to succeed the role of Information Sharing and Analysis Centers and national Computer Emergency Response Teams is critical.

## Conclusions

Cyber insurance is considered a strategy for risk management. It is emerging as an important tool to protect organizations against cyberattack-related losses. In this work we extensively examined the relevant literature on the cyber insurance field, research and practice, to present the current landscape and to provide insights and future directions. The findings and guidelines presented in this paper will be of use for ICT professionals upon considering cyber insurance services as a complementary option for risk management. Current risks driving the cybersecurity market include ransomware, (the increased) remote working, breaches of business email, supply chain and third-party attacks and fraud in the transfer of funds.

This work also provided an overview of cyber insurance practices by analyzing major cybercrime breaches that a cyber insurance contract has to deal with, but also the standard conditions / criteria for a risk to be considered eligible for a cyber claim. Our findings may also serve as guidelines for the private and public sector that are examining cyber insurance, in order to be aware of the current practices of the cyber insurance services, for the management of the effects of cybercrime incidents.

Cyber insurance market growth is in progress, with some aspects being characterized by ambiguity. The field has to tackle with challenges and obstacles which are not typically found in the insurance market, like the correlation of risks and the geographic dispersity of risk. Other organizations do not opt for cyber insurance because of the high cost of contracts, concerns about the insurance coverage and lack of information on the practices and policies of cyber insurance providers. In addition, the complexity of the cyber claims process, following the occurrence of incidents, that could fall within the scope of an insurance policy is a matter of concern. However, data show that the cyber insurance market is gradually maturing and uncertainties are decreasing. At the same time, the rise of advanced cybersecurity attacks is transforming cyber insurance into a critical (additional) component in risk management, in order for an organization to most appropriately respond to modern threats and the significant impact that arises from their implementation.

## Data Availability

This research did not use or generate any data.

## Footnotes

1 https://www.psafinancial.com/cyber-insurance-claim-process/

2 https://www.coalitioninc.com/

3 https://www.at-bay.com/
